# Fixation of carbon dioxide into dimethyl carbonate over titanium-based zeolitic thiophene-benzimidazolate framework

**DOI:** 10.1038/s41598-017-00736-1

**Published:** 2017-04-05

**Authors:** Sanny Verma, R. B. Nasir Baig, Mallikarjuna N. Nadagouda, Rajender S. Varma

**Affiliations:** 1 Oak Ridge Institute for Science and Education, P. O. Box 117, Oak Ridge, TN 37831 USA; 2 WQMB, WSWRD, National Risk Management Research Laboratory, U. S. Environmental Protection Agency, 26 West Martin Luther King Drive, Cincinnati, Ohio 45268 USA; 3 Sustainable Technology Division, National Risk Management Research Laboratory, U. S. Environmental Protection Agency, 26 West Martin Luther King Drive, MS 443, Cincinnati, Ohio 45268 USA

## Abstract

A titanium-based zeolitic thiophene-benzimidazolate framework has been designed for the direct synthesis of dimethyl carbonate (DMC) from methanol and carbon dioxide. The developed catalyst activates carbon dioxide and delivers over 16% yield of DMC without the use of any dehydrating agent or requirement for azeotropic distillation.

## Introduction

Environmental protection has become a challenging task with the growing ecological imbalance and threat posed by climate change^1^. Scientists are looking for alternatives to minimize the growing concentration of carbon dioxide (CO_2_) in the surroundings and curtail the release of greenhouse gases^2^. The ideal solution is to minimize or eliminate the emission of CO_2_ into the atmosphere. In the absence of any meaningful control of the rising level of CO_2_, immediate necessity appears to find a technological solution to capture and convert CO_2_ into useful chemicals feedstocks^3^. Dimethyl carbonate (DMC) is an important industrial compound which is used in methylation chemistry^4^, as a biodegradable organic solvent^5–7^, building blocks in pharmaceutical industries^8–10^ and recognized as a high octane fuel^11^. In view of the growing functions of DMC in industrial products it became imperative to design a simple and sustainable protocol for the synthesis of dimethyl carbonate, preferably using abundant greenhouse gas, CO_2_^12^. Earlier, scientists have used phosgene as starting material in DMC synthesis^13^. In order to eliminate the toxic feedstocks required for DMC synthesis transition metal catalyzed reactions have been developed^14, 15^. The reported methods, however, often require longer reaction time, high temperature and pressure to accomplish the synthesis. Alternatively, oxidative carbonylation of methanol^16, 17^, transesterification of cyclic carbonates^12^, methanolysis of urea^12^ have been deployed but these methods suffer from low yield and diminished atom efficiency resulting in the generation of large amount of chemical waste along with compromised yield.

Carbon dioxide is one of the most basic oxygenated carbon which can be converted into corresponding DMC^15^; several reports document transforming carbon dioxide into DMC^18–20^. However, they invariably suffer from reversibility of the reaction, because water is generated as a byproduct in the reaction mixture which reacts with the product DMC thus reverting it back into starting materials^21^. To carry this reaction forward, an azeotropic distillation is mandatory which is not a trivial proposition to setup under high pressure conditions. Alternatively, water trapping reagents such as molecular sieves need to be added to the mix^22, 23^. Our efforts have focused on finding simple alternative protocols for the synthesis of industrially important products^24–27^. To accomplish a simple synthesis of DMC, we have now designed a titanium-based zeolitic thiophene-benzimidazolate framework (Ti-ZTBF) and demonstrated its application in the chemical fixation of carbon dioxide into DMC without the use of water trapping agents.

## Results and Discussion

**Figure 1:**
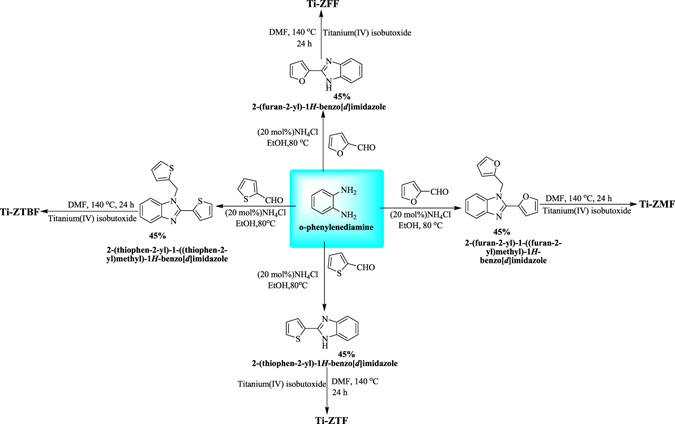
Synthesis of different ligands and Ti-based zeolitic imidazolate frameworks.

We planned to synthesize zeolitic framework and explore its application in DMC synthesis using carbon dioxide and methanol with a keen eye to address the main challenge that has appropriate control over the reversible nature of reaction. We envisioned that metal zeolitic framework would address the problem as the porous nature and high surface area of the material would keep water molecules away from the DMC product thus promoting the equilibrium shift in right direction. We embarked on our study to search for and synthesize a suitable ligand for the creation of zeolitic type metal framework. In our quest, we synthesized four different ligands namely, 2-(furan-2-yl)-1*H*-benzo[d]imidazole,2-(furan-2-yl)-1-((furan-2-yl)methyl)-1*H*-benzo[d]imidazole, 2-(thophen-2-yl)-1*H*-benzo[d]imidazole and 2-(thophen-2-yl)-1-((thiophen-2-yl)methyl)-1*H*-benzo[d]imidazole. These ligands were than treated with titanium iso-butoxide in a pressure reactor at 140 °C using DMF as solvent for 24 hours; the formation of crystalline solid ensued in reaction vessel (see Supplementary). It was then centrifuged, washed with methanol and vacuum dried to form Ti-ZFF (BET surface area = 369.01 m^2^g^−1^), Ti-ZMF (BET surface area = 316.30 m^2^g^−1^), Ti-ZTF (BET surface area = 140.10 m^2^g^−1^) and Ti-ZTBF (BET surface area = 811.36 m^2^g^−1^), respectively (Fig. 1).

After securing the catalyst, we screened these zeolitic framework entities for the conversion of CO_2_ to DMC. Initially, we performed the reactions at different pressure of CO_2_ at room temperature; at 10 and 20 psi none of these catalysts gave any DMC (Table 1, entries 1-8). However, at 30 psi, we observed the trace amounts of DMC in presence of Ti-ZTBF (Table 1, entry 12). Porosity and high surface area of Ti-ZTBF catalyst made it more effective towards the synthesis of DMC. It became apparent that Ti-ZTBF is the effective catalyst for this transformation and we continued forward with our optimization explorations using Ti-ZTBF as a catalyst with appropriate variation in temperature in addition of increasing CO_2_ pressure. The reaction at 50 °C gave 1.5% of DMC which increased to 9% and 16% isolated yield at 75 °C and 100 °C, respectively (Table 1, entries 13-15). A further increase in the temperature to 120 °C by keeping pressure at 30 psi reaction did not improve the output thus affirming that 100 °C appears to be the optimum temperature for this reaction. The effect of increased pressure was also examined at 40 and 50 psi at 100 °C but it did not improve the outcome of these control experiments as the isolated yield of DMC remained stubbornly at 16% (Table 1, entries 17-18). It seems that 30 psi pressure and 100 °C temperature are the ideal conditions for this transformation. Further probing experiments were conducted by varying the catalytic amount of Ti-ZTBF which confirmed a sharp drop in the product yield at 50 mg level. Increasing the concentration of catalyst from 100 mg to 150 mg did not show any significant improvement in the product yield thus establishing that 100 mg is the optimum amount of catalyst required for the successful synthesis of DMC (Table 1, entries 19-20).

### Synthesis and characterization of Ti-ZTBF

**Figure 2:**
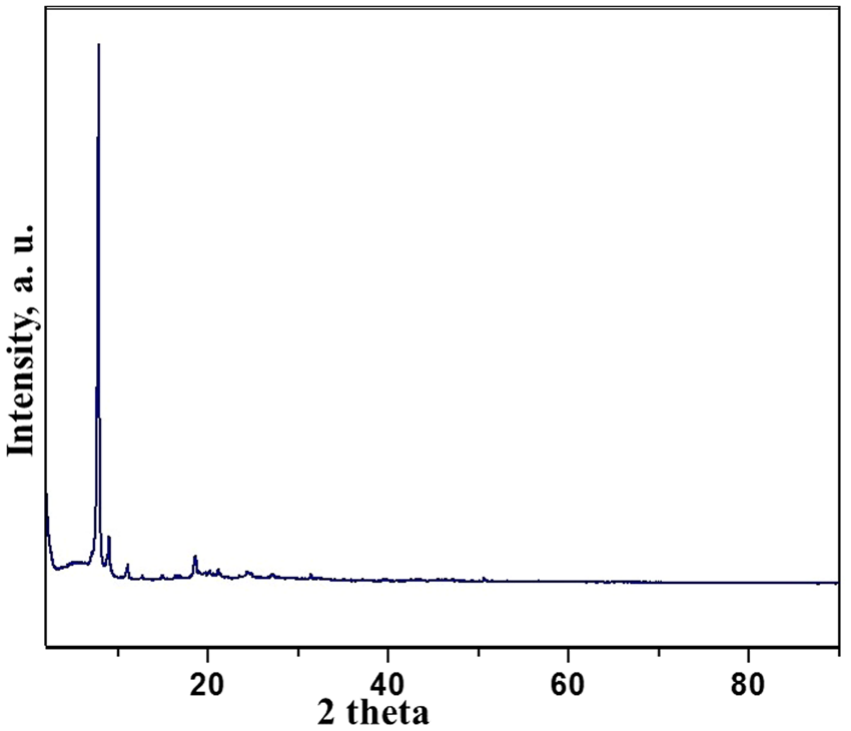
XRD analysis of Ti-ZTBF.

**Figure 3:**
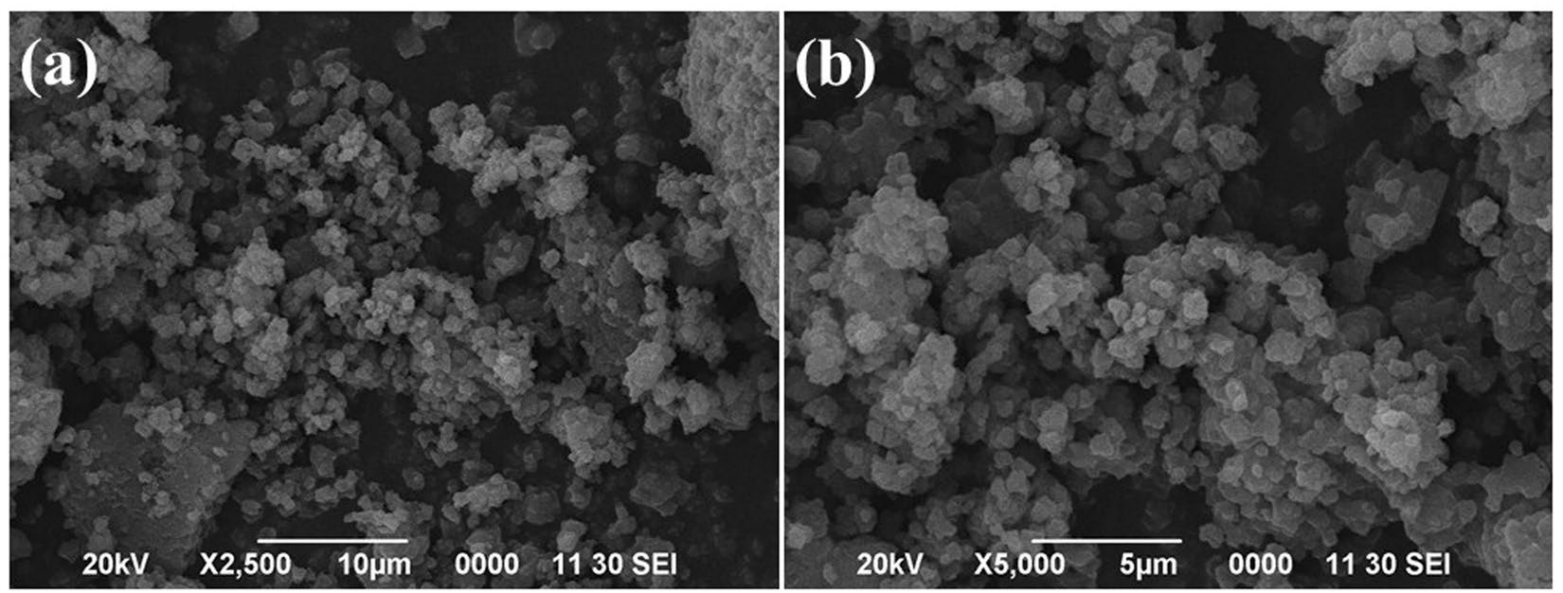
SEM analysis of Ti-ZTBF.

**Figure 4:**
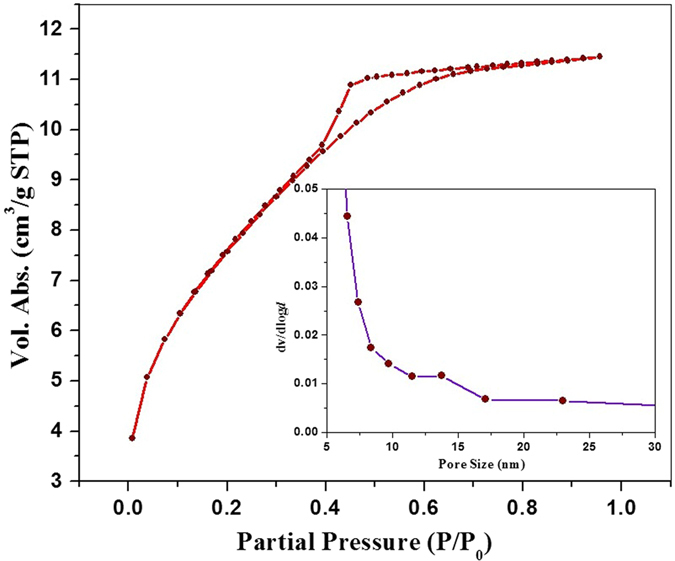
BET analysis of Ti-ZTBF.

**Figure 5:**
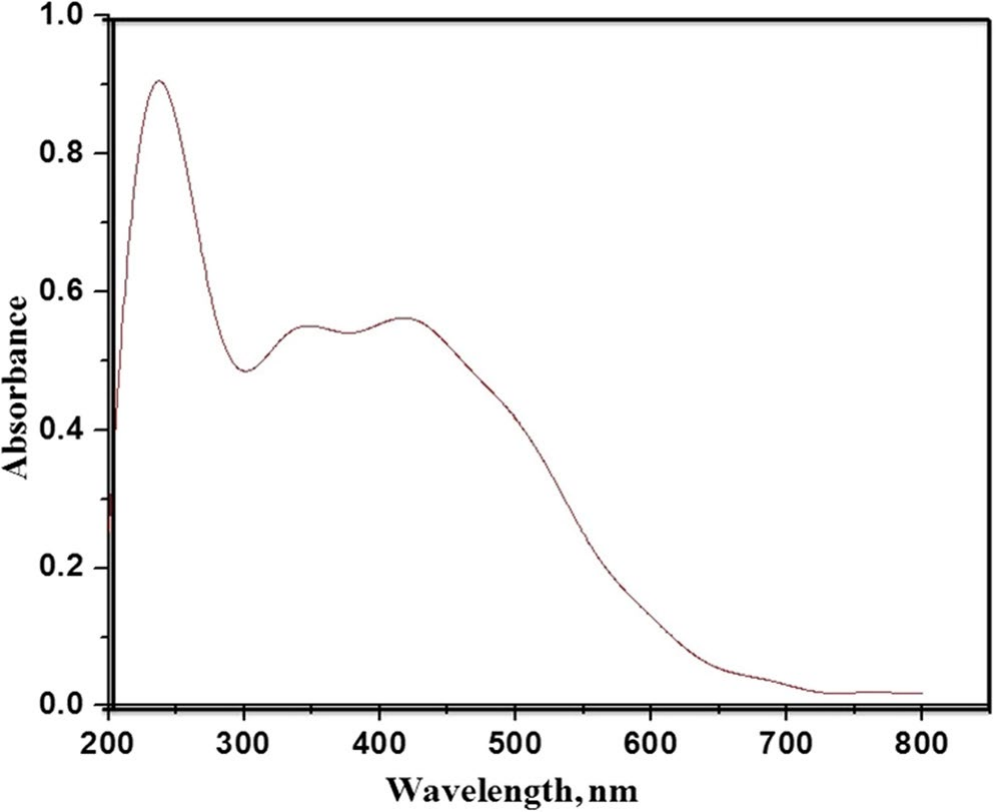
UV-Vis absorption spectra of Ti-ZTBF.

The ligand 2-(thophen-2-yl)-1-((thiophen-2-yl) methyl)-1*H*-benzo*[d]*imidazole was synthesized by treating *o*-phenylenediamine with thiophene-2-carboxyladehyde at 80 °C in ethanol using 20 mol% ammonium chloride (Fig. 1)^28^. The 2-(thophen-2-yl)-1-((thiophen-2-yl) methyl)-1*H*-benzo*[d]*imidazole was than treated with titanium (IV) isobutoxide (C_16_H_36_O_4_Ti) in an autoclave at 150 °C using DMF as the reaction media. The Ti-ZTBF precipitated out in the reaction mixture. The synthesized Ti-ZTBF catalyst was separated by filtration and characterized using scanning electron microscope (SEM), X-ray diffraction (XRD), Brunauer-Emmett-Teller (BET) surface area analysis and UV-Visible spectroscopy. XRD pattern affirmed the crystalline nature (Fig. 2) of the catalyst with a budding flower-type morphology (SEM image, Fig. 3). The BET analysis portrays the desirable traits of porosity and unprecedentedly high surface area (811.36 m^2^/g, Fig. 4). The UV-Vis spectrum of Ti-ZTBF (Fig. 5) shows a strong absorption peak at 231 nm, which is characteristics of the π-π* transition of C-C bonds in 2-(thophen-2-yl)-1-((thiophen-2-yl) methyl)-1*H*-benzo*[d]*imidazole. The shoulder at 330 nm is assigned to n-π* transition of C=N bond and the absorption band range from 300 to 450 nm is accountable for the yellow-orange color of the Ti-ZTBF. The absorption in Ti-ZTBF is induced by LMCT (Ligand to metal charge transfer) and exhibits a wide absorption band in the visible region with the absorption edge covering ~600 nm^29^.

## Conclusions

Titanium-based zeolitic thiophene-benzimidazolate framework (Ti-ZTBF) has been designed and synthesized. Its application has been demonstrated in the activation of carbon dioxide and direct synthesis of dimethyl carbonate from methanol. The porous morphology and high surface area of Ti-ZTBF plays a crucial role in high activity enabling the reaction to process at lower temperature and CO_2_ pressure. The Ti-ZTBF is a unique catalyst developed to date which takes the reaction in forward direction by circumventing the use of dehydrating agent and avoiding elaborative azeotropic distillation set-ups. The developed catalyst shows good recyclability and could be used up to five times without losing its activity.

**Table 1:** Screening of catalysts and reaction optimization for the direct synthesis of DMC^a^.

Entry	Catalyst	CO_2_ pressure (psi)	Temperature (°C)	Yield^b^
1	Ti-ZFF	10	25 °C	—
2	Ti-ZMF	10	25 °C	—
3	Ti-ZTF	10	25 °C	—
4	Ti-ZTBF	10	25 °C	—
5	Ti-ZFF	20	25 °C	—
6	Ti-ZMF	20	25 °C	—
7	Ti-ZTF	20	25 °C	—
8	Ti-ZTBF	20	25 °C	—
9	Ti-ZFF	30	25 °C	—
10	Ti-ZMF	30	25 °C	—
11	Ti-ZTF	30	25 °C	—
12	Ti-ZTBF	30	25 °C	Traces
13	Ti-ZTBF	30	50 °C	1.5%
14	Ti-ZTBF	30	75 °C	9%
15	Ti-ZTBF	30	100 °C	16%
16	Ti-ZTBF	30	120 °C	15.8%
17	Ti-ZTBF	40	100 °C	16%
18	Ti-ZTBF	50	100 °C	15.9%
19^c^	Ti-ZTBF	30	100 °C	11%
20^d^	Ti-ZTBF	30	100 °C	16%

Reaction condition: ^a^Methanol (5.0 ml), catalyst (100 mg); ^b^Isolated yield. ^c^50 mg of Ti-ZTBF; ^d^150 mg of Ti-ZTBF.

## Electronic supplementary material

Fixation of carbon dioxide into dimethyl carbonate over titanium-based zeolitic thiophene-benzimidazolate framework
