# Supplementary material for: Fixation of carbon dioxide into dimethyl carbonate over titanium-based zeolitic thiophene-benzimidazolate framework
Source: Sci Rep. 2017 Apr 5;7:655. doi: 10.1038/s41598-017-00736-1 (PMC5429631; doi:10.1038/s41598-017-00736-1)
Supplement: Supplementary file 1 — Fixation of carbon dioxide into dimethyl carbonate over titanium-based zeolitic thiophene-benzimidazolate framework [file 41598_2017_736_MOESM1_ESM.pdf]

Supporting Information

Fixation of carbon dioxide into dimethyl carbonate over titanium-based zeolitic thiophene-benzimidazolate framework

Sanny Verma[a], R. B. Nasir Baig[a], Mallikarjuna N. Nadagouda[b] and Rajender S. Varma[c]*

[a]Oak Ridge Institute for Science and Education, P. O. Box 117, Oak Ridge TN, 37831, USA.

[b]WQMB, WSWRD, National Risk Management Research Laboratory, U. S. Environmental Protection Agency, 26 West Martin Luther King Drive, Cincinnati, Ohio 45268, USA.

[c]Sustainable Technology Division, National Risk Management Research Laboratory, U. S. Environmental Protection Agency, 26 West Martin Luther King Drive, MS 443, Cincinnati, Ohio 45268, USA. Fax: 513- 569-7677; Tel: 513-487-2701. E-mail: varma.rajender@epa.gov

Synthesis of 2-(thophen-2-yl)-1-((thiophen-2-yl)methyl)-1*H*-benzo[*d*]imidazole

Synthesis of Ti-ZTBF catalyst

Recycling of Ti-ZTBF catalyst

Tentative mechanism for dimethyl carbonate formation from methanol and CO_2

XRD spectra of recycled Ti-ZTBF catalyst

^{1}H and ^{13}C NMR of the product

Synthesis of 2-(thophen-2-yl)-1-((thiophen-2-yl)methyl)-1*H*-benzo[*d*]imidazole

To a mixture of *o*-phenylenediamine (1 mmol; 108 mg) and thiophen-2-carboxyladehhyde (1 mmol; 112 mg) in 5 mL of ethanol, 20 mol % of ammonium chloride was added and the solution was stirred at 80 °C. The progress of reaction was monitored by TLC. After completion of the reaction (6.0 hours), the product was extracted using ethyl acetate, dried over sodium sulfate and concentered under reduced pressure. The product was purified using column chromatography (ethyl acetate: hexane, 1:1) and characterized using GC-MS.

Synthesis of Ti- ZTBF catalyst

Titanium (IV) isobutoxide (2.0 mmol; 680.5 mg), 2-(thophen-2-yl)-1-((thiophen-2-yl)methyl)-1H-benzo*[d]*imidazole (1 mmol; 296 mg) and dimethylformamide (50 ml) were charged in a pressure reactor. The mixture was heated in 140 °C in an oven for 24 hours. After 24 hours, the reaction was allowed to come down to room temperature. An off-white solid appears in the reaction mixture which was isolated using centrifugation.

3. Recycling of Ti-ZTBF catalyst:

After the completion of each reaction, the Ti-ZTBF catalyst was recovered using a centrifuge, washed with water, dried under vacuum and used for a fresh set of reactants. It was observed that the catalyst remains active even after fifth cycle and could be reused several times without losing activity.

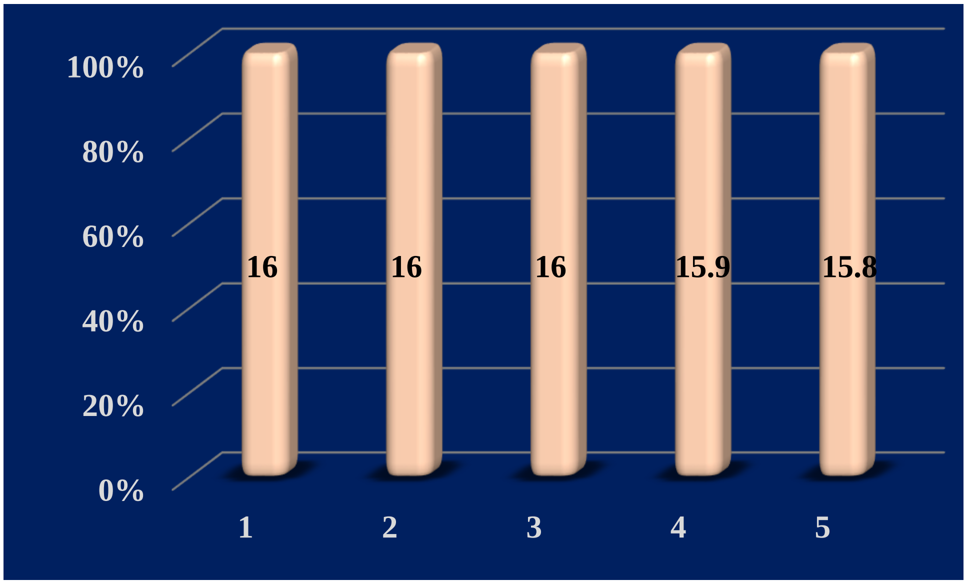

S1. Recycling of Ti-ZTBF catalyst

Tentative mechanism for dimethyl carbonate formation from methanol and CO_2

A tentative mechanism is proposed for the synthesis of DMC formation from methanol and CO_2 (S2). Ti-ZTBF catalyst has both, acidic and basic sites which are important factors for the acceleration of the reaction as shown below.

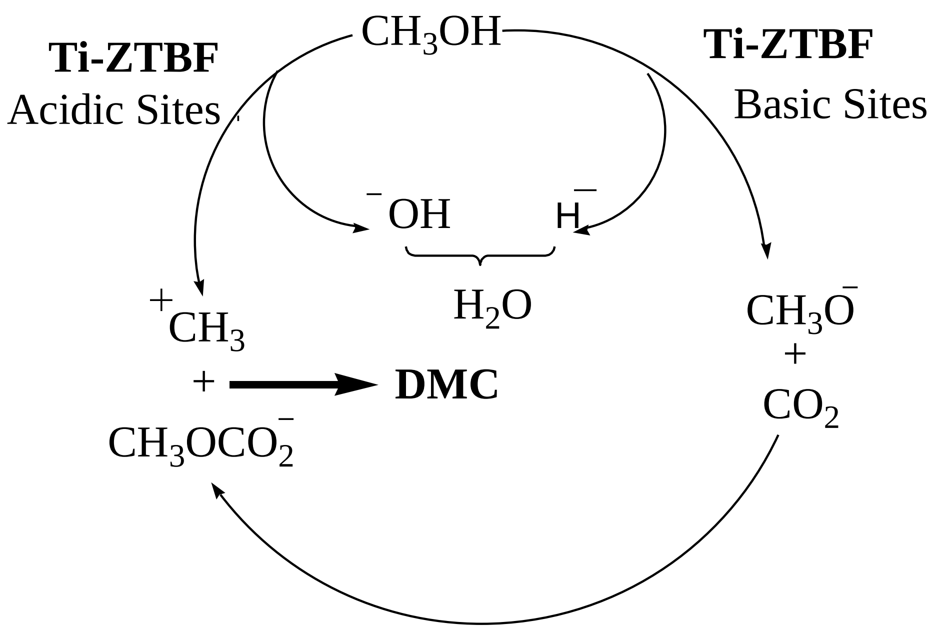

S2. Tentative mechanism for DMC formation from methanol and CO_2

3. XRD spectra of recycled Ti-ZTBF catalyst

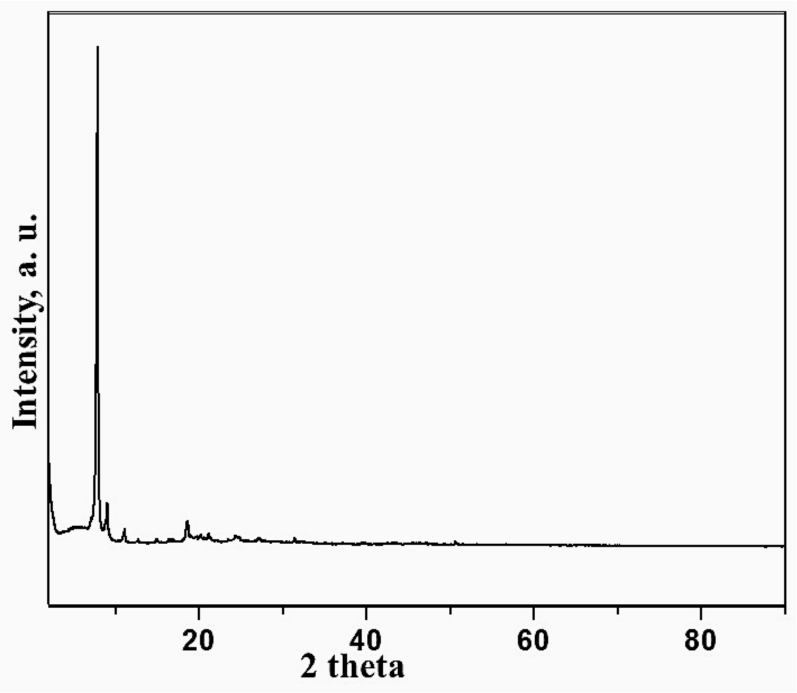

S3. XRD spectra of recycled Ti-ZTBF catalyst

^{1}H and ^{13}C NMR of the product

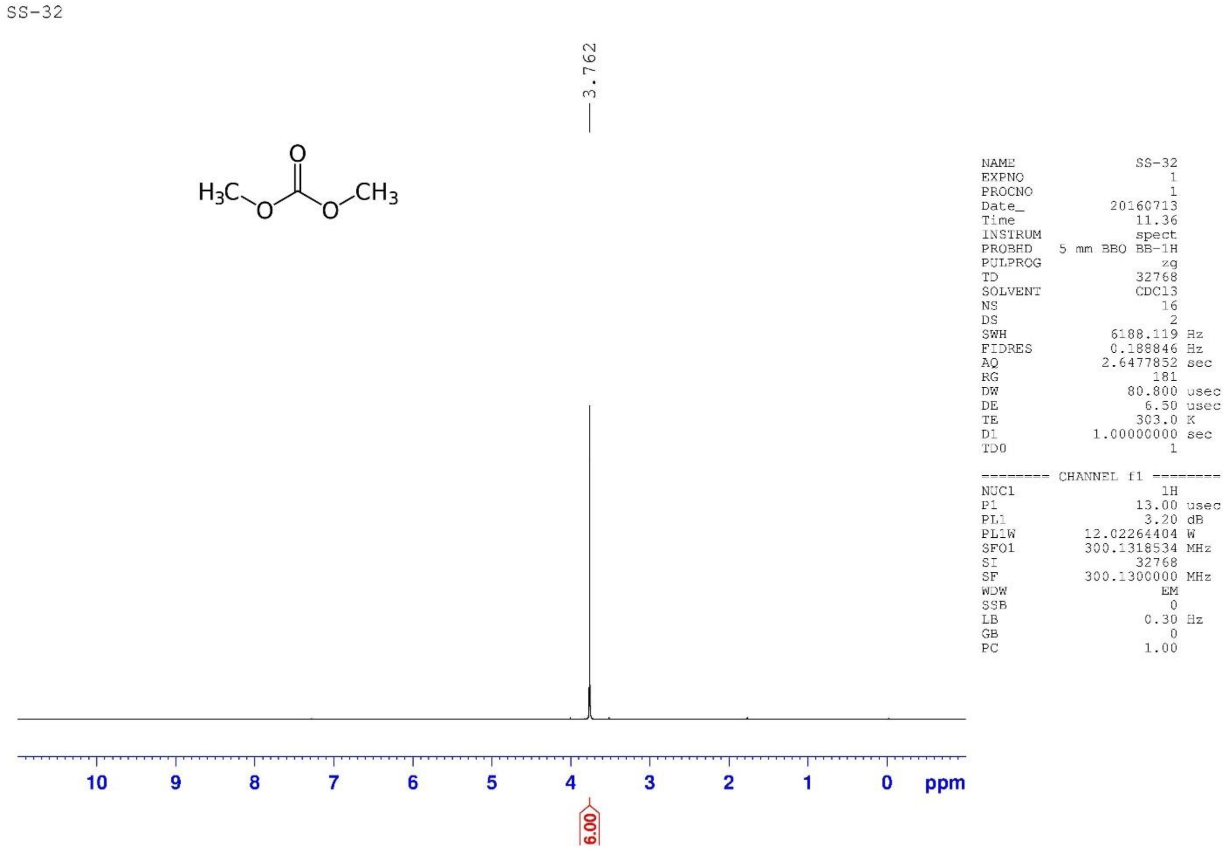

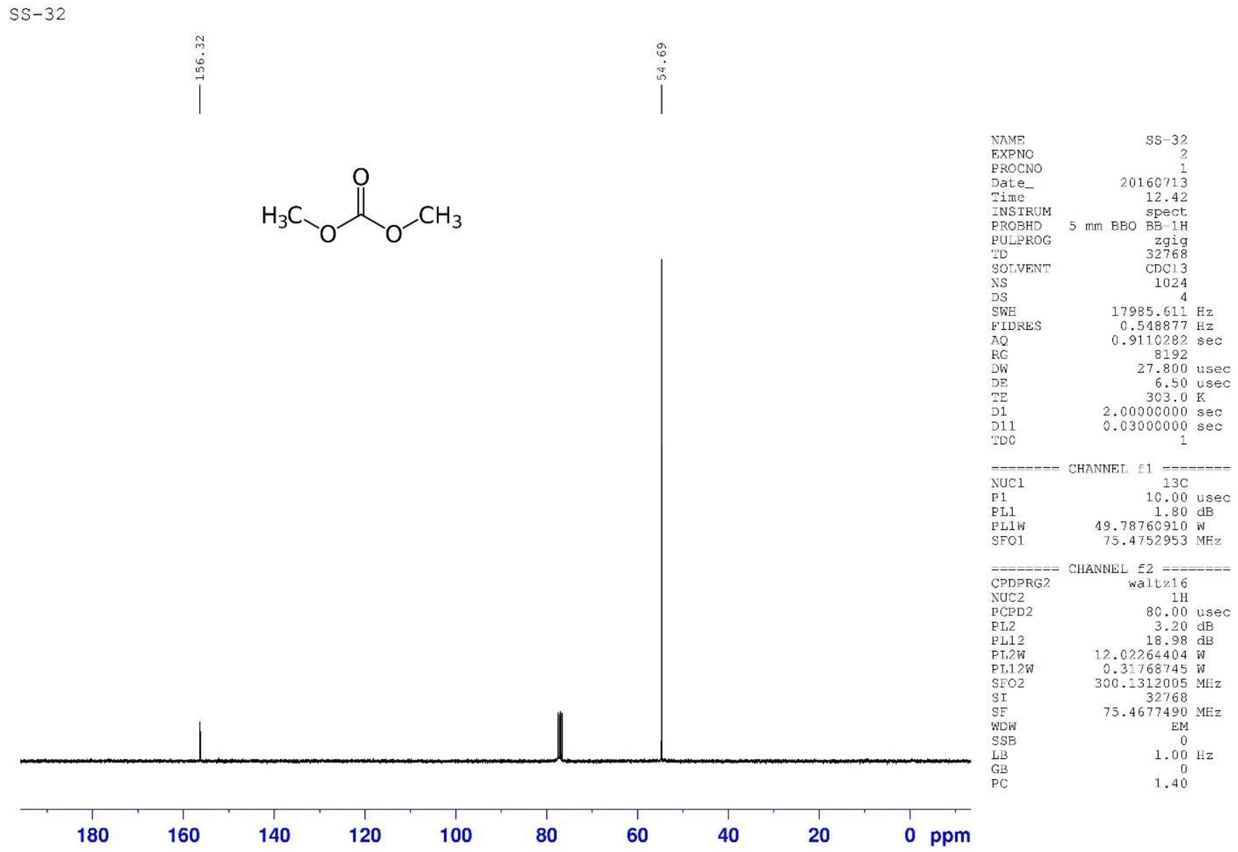
SS-32
156.32
54.69
O
H3C O O CH3
NAME SS-32
EXPNO 2
PROCNO 1
Date_ 20160713
Time 12.42
INSTRUM spect
PROBHD 5 mm BBO BB-1H
PULPROG zgig
TD 32768
SOLVENT CDCl3
NS 1024
DS 4
SWH 17985.611 Hz
FIDRES 0.548877 Hz
AQ 0.9110282 sec
RG 8192
DW 27.800 usec
DE 6.50 usec
TE 303.0 K
D1 2.00000000 sec
D11 0.03000000 sec
TD0 1
======== CHANNEL f1 ========
NUC1 13C
P1 10.00 usec
PL1 1.80 dB
PL1W 49.78760910 W
SFO1 75.4752953 MHz
======== CHANNEL f2 ========
CPDPRG2 waltz16
NUC2 1H
PCPD2 80.00 usec
PL2 3.20 dB
PL12 18.98 dB
PL2W 12.02264404 W
PL12W 0.31768745 W
SFO2 300.1312005 MHz
SI 32768
SF 75.4677490 MHz
WDW EM
SSB 0
LB 1.00 Hz
GB 0
PC 1.40
180 160 140 120 100 80 60 40 20 0 ppm
